# Microfluidic methacrylated hyaluronic acid microspheres incorporating MnO_2_ and exosomes for antioxidant defense and inflammation regulation in osteoarthritis

**DOI:** 10.1016/j.mtbio.2026.103300

**Published:** 2026-05-29

**Authors:** Jianxiang Teng, Jiazhao Pan, Pengcheng Yan, Tianqi Zhou, Zihao Zou, Xiaolin Shu, Hao Liu, Zhuoran Zhang, Sheng Zhou, Qiuhan Chen, Dandan Shen, Xing Zhao, Xiaobin Tian, Long Yang

**Affiliations:** a Department of Orthopedics, The Affiliated Hospital of Guizhou Medical University, China; b Center for Tissue Engineering and Stem Cell Research, Translation Medicine Research Center, Guizhou Biomanufacturing Laboratory, Guizhou Medical University, China; c School of Basic Medical Sciences, Guizhou Medical University, China; d Guizhou StarBio Therapeutics Technology Co., Ltd, China

**Keywords:** Osteoarthritis, Microfluidics, Nanozyme, Exosomes, Methacrylated hyaluronic acid, Mitochondrial function

## Abstract

Osteoarthritis (OA) is a degenerative joint disease characterized by cartilage degeneration, chronic inflammation, and oxidative stress-induced mitochondrial dysfunction. Herein, we engineered an integrated therapeutic platform by encapsulating mesenchymal stem cell-derived exosomes (Exo) and manganese dioxide (MnO_2_) nanozymes into methacrylated hyaluronic acid (HAMA) hydrogel microspheres (HAMA-Exo-MnO_2_) via microfluidic fabrication. Systematic in vitro and in vivo evaluations assessed the microspheres' anti-inflammatory, antioxidant, and chondrogenic regenerative properties. In vitro experiments demonstrated that HAMA-Exo-MnO_2_ microspheres effectively attenuated OA-like pathological changes in chondrocytes, remodeled the inflammatory microenvironment, and scavenged reactive oxygen species (ROS). In a rat OA model induced by destabilization of the medial meniscus (DMM), intra-articular injection of HAMA-Exo-MnO_2_ microspheres ameliorated disease progression, enhanced articular cartilage regeneration, and restored joint function. Transcriptomic sequencing and subsequent validation in cocultured OA chondrocytes revealed significant upregulation of ALDH3A1, which mitigated oxidative stress, activated the NRF2 signaling axis, promoted downstream antioxidant gene expression, and restored mitochondrial electron transport chain activity. In summary, this study highlights that the engineered HAMA hydrogel microspheres provide a multifunctional therapeutic strategy for OA treatment, integrating immune microenvironment modulation, cartilage regeneration, and antioxidant protection.

## Highlights

• Methacrylated hyaluronic acid enables microfluidic fabrication of microspheres.
• These microspheres enable synergistic MnO_2_ and exosome co-delivery.
• The polysaccharide platform modulates immune responses and scavenges ROS.
• ROS scavenging and ALDH3A1/NRF2/activation restore mitochondrial ETC function.
• The platform integrates antioxidant and chondrogenic activities for OA therapy.

## Graphical abstract

Schematic illustration of the synthesis and function of HAMA-Exo-MnO_2_ hydrogel microspheres.

**Figure:**
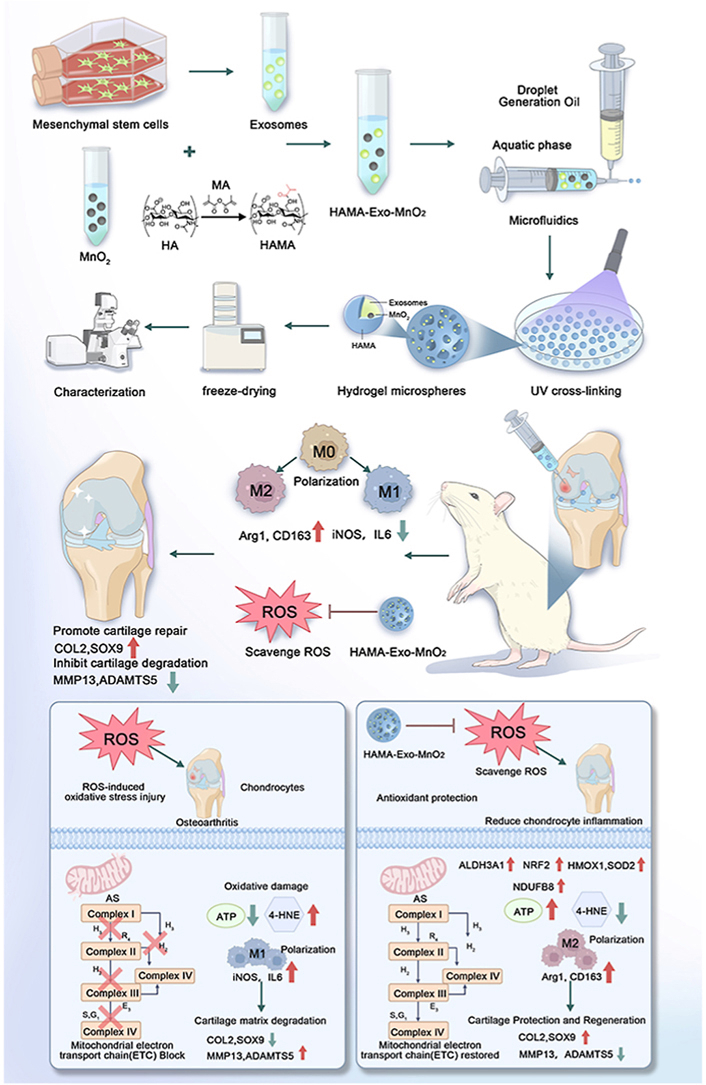

## 1 Introduction

Osteoarthritis (OA) is a common degenerative joint disease affecting more than 500 million people worldwide [1,2]. It is characterized by progressive articular cartilage degradation, synovial inflammation, aberrant remodeling of subchondral bone, and joint space narrowing [3,4]. Articular cartilage exhibits low metabolic activity, with the extracellular matrix (ECM) constituting 95%-99% of its composition, while chondrocytes account for only 1%-5% [5,6]. Furthermore, native cartilage lacks vascular, neural, or lymphatic supply systems, strictly limiting its intrinsic regenerative capacity [6]. Existing drug delivery strategies for early-stage OA commonly suffer from low drug utilization rates, rapid intra-articular clearance, and limited joint retention [7,8]. Their therapeutic outcomes often fall short of expectations, failing to effectively reverse the progressive worsening of OA. This ultimately leads to end-stage surgical interventions such as high tibial osteotomy, unicompartmental knee arthroplasty, or total knee arthroplasty [9]. However, complications arising from surgery and the limited lifespan of prostheses may significantly diminish long-term therapeutic efficacy, highlighting the urgent need for advanced, non-surgical regenerative platforms [10,11].

In recent years, extensive research has demonstrated that excessive production of reactive oxygen species (ROS) is a key driver in the pathogenesis of OA [12,13]. ROS not only induce chondrocyte apoptosis and release inflammatory mediators but also disrupt mitochondrial electron transport chain (ETC) function, further exacerbating oxidative damage and energy metabolism imbalance, thereby creating a vicious cycle [[14], [15], [16]]. Studies have demonstrated significantly elevated levels of ROS and their downstream products (e.g., 4-HNE) in the synovial fluid and serum of OA patients, accompanied by markedly reduced antioxidant capacity (e.g., GSH, SOD) [17]. However, traditional antioxidant therapeutic strategies often face multiple challenges, including limited disease-site specificity, narrow therapeutic windows, and difficulties in simultaneously regulating the inflammatory microenvironment [18]. To improve oxidative damage and the chronic inflammatory microenvironment in OA, developing novel drug delivery systems that enable localized ROS scavenging and regulate the local inflammatory microenvironment is essential for achieving precision and efficacy in OA treatment [19].

Although traditional nonsteroidal anti-inflammatory drugs and intra-articular lubricant injections can alleviate symptoms to some extent, these strategies generally suffer from poor targeting, low bioavailability, narrow therapeutic windows, and difficulties in simultaneously regulating the inflammatory microenvironment and promoting cartilage regeneration [20,21]. Hyaluronic acid (HA), a primary component of the joint extracellular matrix, has long been utilized as a clinical benchmark for OA therapy due to its excellent biocompatibility and lubrication properties [22]. However, native HA is susceptible to rapid enzymatic degradation by hyaluronidase, leading to limited residence time within the joint space [[23], [24], [25]]. Methacrylated hyaluronic acid (HAMA) represents a typical modification that preserves the innate biological cues of HA while introducing photocrosslinkable moieties [26]. Compared to unmodified polysaccharides, HAMA allows for the precise engineering of hydrogel microspheres via microfluidics, providing superior mechanical stability and tunable degradation profiles essential for sustained drug release [27]. Manganese-based nanoparticles (e.g., MnO_2_) exhibit potent ROS scavenging capabilities in various oxidative stress-related diseases due to their exceptional peroxidase-like activity, which efficiently catalyzes H_2_O_2_ decomposition into water and oxygen [28]. Exosomes (Exo) derived from mesenchymal stem cells are rich in bioactive components and exhibit significant chondrogenic and immunomodulatory effects, including the ability to induce macrophage polarization toward the anti-inflammatory M2 phenotype [[29], [30], [31], [32]]. However, constructing a localized delivery platform with dual-functional capabilities that simultaneously achieves ROS scavenging and immune regulation remains a current research challenge. The combined delivery of MnO_2_ and Exo holds promise for achieving synergistic effects in antioxidant defense and regenerative repair [33,34].

Recently, functional hydrogel microspheres have shown remarkable progress in facilitating cartilage healing, mediating tissue signal transduction, and alleviating osteoarthritis pain [35]. Here, we report a microfluidic-fabricated HAMA hydrogel-based delivery platform (HAMA-Exo-MnO₂) as a functional therapeutic vehicle. This platform co-delivers stem cell–derived exosomes and MnO_2_ nanozyme particles to mitigate cartilage degeneration and improve knee function through multi-pronged actions, including ROS scavenging, macrophage M2 polarization, and promotion of chondrocyte regeneration. Mechanistically, transcriptomic profiling further revealed a marked upregulation of the antioxidant-related gene ALDH3A1 and activation of the NRF2 signaling axis, consistent with restored mitochondrial function and improved cartilage matrix homeostasis. Collectively, this work provides an integrated antioxidant/regenerative strategy for OA treatment with potential translational relevance, leveraging methacrylated hyaluronic acid (HAMA) hydrogel microspheres as a functional therapeutic vehicle.

## 2 Materials and methods

### 2.1 Materials and reagents

Methacrylated hyaluronic acid (HAMA, molecular weight 100-200 kDa, degree of substitution 60-70%, purity≥96%) was purchased from Shenzhen Shenning Biotechnology Co., Ltd. (Shenzhen, China). Potassium permanganate (KMnO_4,_ purity≥99.0%) was obtained from Sigma-Aldrich (St. Louis, MO, USA). Hydrogen peroxide (H_2_O_2_, 30% w/w) was purchased from Merck KGaA (Darmstadt, Germany). The photoinitiator lithium phenyl-2,4,6-trimethylbenzoylphosphinate (LAP, purity 98%) was obtained from Engineering for Life (Suzhou, China).

### 2.2 Preparation and characterization of mesenchymal stem cell-derived exosomes

Human umbilical cord mesenchymal stem cells (hUC-MSCs) were kindly provided by Prof. Xing Zhao (Tissue Engineering and Stem Cell Experiment Center, Guizhou Medical University). Exosomes were isolated from hUC-MSC conditioned medium as follows: Cells were cultured in serum-free, exosome-depleted medium for 48 h. The conditioned medium was collected and centrifuged at 2000 × g for 20 min at 4 °C to remove cells and large debris, followed by filtration through a 0.22 μm membrane. The clarified supernatant was subsequently processed using an ultrafast isolation system (EXODUS, Huixin Lifetech, Shenzhen, China), which integrates acoustic focusing with nanofluidic filtration to enable rapid, automated enrichment of exosomes in a closed and contamination-controlled workflow. The isolated exosomes were stored at −80 °C until further characterization and use.

### 2.3 Preparation of nanoscale MnO_2_

Nanoscale manganese dioxide (MnO_2_) was synthesized via a redox reaction. Briefly, 50 mL of 0.1 M potassium permanganate (KMnO_4_) solution was mixed with 10 mL of 30% (w/w) hydrogen peroxide (H_2_O_2_) in a 250 mL conical flask at 25 ± 2 °C. The mixture was stirred at 600 rpm for 30 min until the solution turned dark brown, indicating MnO_2_ formation. The product was collected by centrifugation at 15,000 rpm for 15 min and washed three times sequentially with deionized water and anhydrous ethanol. The precipitate was dried in a vacuum oven at 80 °C for 12 h to obtain a dark brown powder. The morphology and dispersion state of MnO_2_ nanoparticles were examined by transmission electron microscopy (TEM). For cytocompatibility screening, MnO_2_ was incubated with chondrocytes at concentrations of 10–200 μg/mL, and cell proliferation was assessed by CCK-8. A concentration of 100 μg/mL showed optimal performance (Supplementary Fig. S1) and was therefore used for subsequent microsphere preparation.

### 2.4 Preparation of HAMA-Exo-MnO_2_ hydrogel microspheres

HAMA was dissolved in phosphate-buffered saline (PBS) to 4 wt%, followed by addition of LAP and MnO_2_. The mixture was sonicated on ice for 30 min to ensure homogeneous dispersion. After sterilization by filtration through a 0.22 μm filter, the HAMA/LAP/MnO_2_ solution was mixed with an exosome suspension under a laminar flow hood to obtain a final exosome concentration of 1 × 10^10^ particles/mL, yielding the precursor solution. This mixture was exposed to 365 nm UV light to achieve complete crosslinking and evaluate its photocrosslinking behavior.

Hydrogel microspheres were fabricated using a droplet-generating polydimethylsiloxane (PDMS) microfluidic chip equipped with a flow-focusing cross-junction (channel inner diameter: 100 μm; Pengzan Biotechnology, Shanghai, China). The aqueous phase consisted of 4 wt% HAMA, LAP, 100 μg/mL MnO_2_, and 1 × 10^10^ particles/mL exosomes. Droplets were generated at a constant oil-to-aqueous flow-rate ratio of 3:1 (oil phase: 12 μL/min; aqueous phase: 4 μL/min) and subsequently crosslinked via 365 nm UV irradiation to yield HAMA-Exo-MnO_2_ microspheres. Following demulsification, the microspheres were thoroughly washed with sterile deionized water to eliminate residual oil. HAMA and HAMA-Exo microspheres were prepared under identical conditions as controls. To evaluate injectability, the microspheres were resuspended in PBS, loaded into a standard 1 mL syringe, and extruded.

### 2.5 Morphological observation of hydrogel microspheres

Microspheres were dispersed in PBS, placed onto glass slides, and imaged using an inverted microscope (Nikon, Japan) to evaluate their morphology. To determine the particle size distribution, microspheres (n = 100) were randomly selected from the bright-field images and their diameters were measured using ImageJ software. The particle size data were statistically analyzed and expressed as mean ± SD. After freeze-drying, the microsphere surface morphology and internal microstructure were examined by scanning electron microscopy (SEM; Nova Nano SEM 230, Japan). Energy-dispersive X-ray spectroscopy (EDS) was performed to map the Mn element distribution.

### 2.6 Composition analysis of hydrogel microspheres

Surface elemental composition was analyzed by X-ray photoelectron spectroscopy (XPS; K-Alpha, USA) using a spot size ≥400 μm. Chemical structures were further examined by Fourier transform infrared spectroscopy (FTIR; Nicolet iS20, Thermo Scientific, USA) in ATR mode. Spectra were collected over 4000–500 cm^−1^ with a resolution of 4 cm^−1^.

### 2.7 Sustained-release properties of microspheres

Prior to encapsulation, exosomes were labeled with the fluorescent dye DiD. The microspheres (100 mg; HAMA-Exo and HAMA-Exo-MnO_2_) were immersed in 1 mL of PBS (pH 7.4, containing 1% PMSF) and incubated at 37 °C under continuous shaking in the dark. At predetermined time points (days 1, 2, 3, 5, 7, 9, 12, 15, and 18), 200 μL of the supernatant was collected and replenished with an equal volume of fresh PBS. Exosome release was quantified by measuring the fluorescence intensity (Ex/Em = 633/665 nm) via a microplate reader to calculate the cumulative release percentage.

For the Mn release assay, HAMA-Exo-MnO_2_ microspheres were incubated in 1 mL of HEPES-NaCl buffer (pH 7.4) at 37 °C with continuous shaking. At the aforementioned time points, 100 μL of the supernatant was sampled and replaced with fresh buffer. The Mn concentration in the collected aliquots was directly quantified using inductively coupled plasma mass spectrometry (ICP-MS) to determine the cumulative Mn release percentage.

### 2.8 Evaluation of catalytic activity and H_2_O_2_ scavenging efficiency

Microspheres (HAMA, HAMA-Exo, and HAMA-Exo-MnO_2_) were incubated in a H_2_O_2_ solution at room temperature for 2 h (Fig. S2). To assess catalytic activity, the concentration of generated dissolved oxygen in the solution was directly recorded using a dissolved oxygen meter. To evaluate the H_2_O_2_ scavenging efficiency, the residual H_2_O_2_ content in the supernatant was quantified using a commercial Hydrogen Peroxide Assay Kit (BC3595, Solarbio, Beijing, China). Briefly, the supernatant was collected and reacted with the kit reagents according to the manufacturer's instructions. The absorbance of the mixture was then measured at 415 nm using a microplate reader, and the H_2_O_2_ scavenging efficiency was calculated accordingly.

### 2.9 Swelling ratio

Lyophilized microspheres (HAMA, HAMA-Exo, and HAMA-Exo-MnO_2_) were accurately weighed to record the initial dry weight (Wd) and then immersed in PBS (pH 7.4) at 37 °C. After 24 h of incubation to reach swelling equilibrium, the microspheres were collected and gently blotted with filter paper to remove excess surface water. The swollen weight (Ws) was immediately recorded. The swelling ratio was calculated using the following formula: (Ws - Wd)/Wd × 100%.

### 2.10 Isolation and characterization of primary human chondrocytes

Knee cartilage specimens were obtained from patients undergoing total knee arthroplasty. This study was approved by the Human Research Ethics Committee of the Affiliated Hospital of Guizhou Medical University (Approval No. 2025-272). Samples were transferred to a biosafety cabinet immediately after collection and rinsed thoroughly with sterile PBS. Cartilage was dissected, minced into ∼1 mm^3^ fragments, and digested with collagenase II (1 mg/mL) at 37 °C for 12 h under shaking. The digestion mixture was centrifuged at 1200 rpm for 3 min, and cells were resuspended in DMEM/F12 supplemented with 10% fetal bovine serum and 1% penicillin/streptomycin, then cultured in T25 flasks. Medium was changed every 2–3 days. Passage-2 chondrocytes were used in subsequent experiments. Toluidine blue and Alcian blue staining were performed for phenotypic identification, and images were acquired using an inverted microscope.

### 2.11 Chondrocyte uptake of exosomes

Exosomes were labeled with DiD and co-incubated with chondrocytes for 12 h. Cells were then washed, fixed as needed, and stained with FITC-phalloidin to visualize F-actin, followed by DAPI nuclear staining. Exosome uptake was observed using an inverted fluorescence microscope.

### 2.12 Biocompatibility assessment of microspheres

A Transwell co-culture system was established to evaluate the effect of microspheres on the biological behavior of chondrocytes. Microspheres were placed in the upper chamber, while human chondrocytes were seeded in the lower chamber and cultured until appropriate confluence.

### 2.13 Cell viability assessment

Chondrocytes were seeded at 5 × 10^4^ cells/well in 12-well plates. After cell adhesion, Transwell chambers containing microspheres were placed into wells, with sufficient medium to fully immerse the microspheres. After 72 h, cells were stained using a Calcein-AM/PI kit (Jiangsu Kaiji) per the manufacturer's instructions, imaged by fluorescence microscopy (Zeiss), and quantified using ImageJ (n = 5).

### 2.14 Cell proliferation assessment

Cells were cultured as described above. After 48 h of co-culture, proliferation was evaluated using an EdU kit (CX003, CellorLab, Shanghai, China). Images were acquired by fluorescence microscopy and quantified using ImageJ (n = 5).

### 2.15 Cell migration assay

Wound healing assay**:** Chondrocytes were seeded at 2 × 10^5^ cells/well in 6-well plates. At ∼90% confluence, scratches were generated using a 200 μL pipette tip. Images were collected at 0 h, then the medium was replaced with DMEM/F12 containing 5% FBS. Transwell chambers containing microspheres were added, and images were recorded again at 24 h to calculate wound closure using ImageJ (n = 5).

Transwell migration assay: Chondrocytes (5 × 10^4^ cells/well) were seeded in the upper chambers in serum-free DMEM/F12, while the lower chambers contained DMEM/F12 with 5% FBS and microspheres from each group. After 24 h, migrated cells were fixed and stained with 0.1% crystal violet, imaged, and quantified using ImageJ (n = 5).

### 2.16 OA-like chondrocyte model and treatment

Chondrocytes were stimulated with LPS (10 μg/mL) for 12 h to establish an OA-like inflammatory model. A microsphere co-culture system was then set up as mentioned above. After 72 h, Alcian blue staining was performed to evaluate acidic mucopolysaccharide deposition. *RT-qPCR*, immunofluorescence, and Western blot were used to assess OA-related pathological changes after treatment.

### 2.17 Antioxidant property evaluation

Chondrocytes were seeded at 5 × 10^4^ cells/well in 12-well plates and allowed to adhere. Cells were then treated with H_2_O_2_ (50 μM) and co-cultured with microspheres for 24 h. Intracellular ROS levels were measured using a ROS assay kit (S1105S, Beyotime, Shanghai, China), imaged by fluorescence microscopy, and quantified by ImageJ (n = 5).

### 2.18 Evaluation of macrophage polarization

Macrophage polarization was assessed using **RAW264.7** cells. For M1 polarization, cells were pretreated with high-glucose DMEM containing LPS (1 μg/mL) for 24 h. For M2 polarization, cells were pretreated with high-glucose DMEM containing IL-4 (20 ng/mL) for 24 h. Cells were then co-cultured with microspheres for 48 h, followed by *RT-qPCR*, immunofluorescence, and Western blot. Primer sequences are provided in Supplementary Table S1, and antibody information is listed in Supplementary Table S2.

### 2.19 RT-qPCR

Total RNA was extracted using TRIzol. cDNA was synthesized using a reverse transcription kit (KR118, Tiangen, Beijing, China). qPCR was performed using Fast Taq SYBR Green (500-102A, Gooniebio, Guangzhou, China). Relative mRNA expression was calculated by the 2^−ΔΔCt^ method. Primer sequences are provided in Supplementary Table S1.

### 2.20 Immunofluorescence

Cells (1 × 10^5^ cells/well) were seeded in 12-well plates and treated as described above. After 48 h, cells were fixed with paraformaldehyde for 10 min, washed with PBS, permeabilized with Triton X-100 for 15 min, and blocked with 3% BSA for 1 h. Cells were incubated with primary antibodies overnight at 4 °C, followed by fluorescent secondary antibodies for 1 h in the dark. F-actin was stained with FITC-phalloidin, and nuclei were counterstained with DAPI. Antibody details are provided in Supplementary Table S2.

### 2.21 Western blot (WB)

Cells were seeded at 2 × 10^5^ cells/well in 6-well plates and treated as described above. After 72 h, total proteins were extracted using RIPA lysis buffer (Solarbio, Beijing, China) containing 1% PMSF on ice. Equal amounts of protein (30 μg) were separated by SDS-PAGE (90 V for 30 min, then 120 V for 80 min) and transferred to membranes. Membranes were blocked with a protein-free rapid blocking solution for 1 h, washed with TBST, and incubated with primary antibodies overnight at 4 °C. After washing, membranes were incubated with HRP-conjugated secondary antibodies for 1.5 h at room temperature. Bands were visualized using an enhanced chemiluminescence system (Clinx, Shanghai, China) and quantified using ImageJ (n = 3).

### 2.22 Sustained retention effect of exosomes in the joint cavity

DiD-labeled exosomes, either free or encapsulated in microspheres (HAMA-Exo and HAMA-Exo-MnO_2_), were intra-articularly injected into rats. At days 0, 3, 6, 9, and 12 post-injection, fluorescence signals from the knee joints of anesthetized rats were captured using an in vivo imaging system. The relative fluorescence intensities were then quantified to evaluate the sustained retention of the materials in the joint cavity.

### 2.23 SD rat OA model

OA was induced in male Sprague–Dawley (SD) rats (∼250 g) using destabilization of the medial meniscus (DMM). All animal procedures were approved by the Laboratory Animal Ethics Committee of Guizhou Medical University (Approval No. 2502971). Briefly, rats (n = 20) were anesthetized with isoflurane, the surgical site was disinfected, and a ∼1.5 cm medial parapatellar incision was made. The patella was displaced laterally to expose the joint, and the anterior horn of the medial meniscus was resected to induce instability. The incision was closed in layers (Fig. S4A, B). Sham-operated rats (n = 5) underwent arthrotomy without meniscus resection. Postoperatively, penicillin (100,000 U) was administered intramuscularly once daily for 5 days, and parecoxib was used for analgesia. Animals had free access to food and water.

### 2.24 Intra-articular injection

Four weeks after surgery, DMM rats (n = 20) were randomly assigned to four treatment groups: Saline, HAMA, HAMA-Exo, and HAMA-Exo-MnO_2_. To ensure sufficient localized delivery of the microsphere suspension in a single dose and minimize the need for repeated administrations, each rat received a 100 μL intra-articular injection of the corresponding formulation [36]. The administration was carefully performed at a slow rate using a fine needle to avoid obvious leakage and excessive resistance. Sham rats received no injection.

### 2.25 Functional assessment

Six weeks after injection, pain thresholds were evaluated using mechanical stimulation and hot-plate tests. For the hot-plate test (Fig. S5A), the plate was preheated to 55 °C and the latency to limb withdrawal was measured by a cold-hot plate pain meter (ZS-CTE, Zhongshi, Beijing, China) (n = 5). For mechanical stimulation (Fig. S5B), the plantar surface of the affected limb was stimulated by electronic von Frey Anesthesiometer (Life Science lnc, USA), and the withdrawal threshold was recorded. For gait analysis (Fig. S5C), forepaws and hindpaws were stained with red and black ink, respectively, and rats traversed a 100 × 5 cm corridor lined with A4 paper. Clear footprints were analyzed by ImageJ to quantify stride length, step width, and fore–hind print overlap (n = 5).

### 2.26 Radiological evaluation

At 8 weeks after injection, knee joints were harvested, fixed in 4% paraformaldehyde, and scanned by X-ray and micro-CT. Three-dimensional reconstructions were used to quantify BV/TV, trabecular thickness (Tb.Th), and trabecular separation (Tb.Sp) (n = 5).

### 2.27 Histological analysis

After micro-CT, cartilage samples were decalcified in 10% EDTA at 37 °C with shaking for 4 weeks, and the solution was replaced every 3 days. Sections were stained with H&E and Safranin O–fast green. Cartilage thickness was measured using ImageJ, and pathological changes were graded using the Mankin scoring system (n = 5). For immunohistochemistry, sections were incubated with a rabbit polyclonal antibody against COL2A1 overnight at 4 °C, followed by secondary antibody incubation for 1 h and DAB development. For immunofluorescence, MMP13 expression was evaluated using a rabbit polyclonal antibody and a fluorescent secondary antibody. Antibody details are provided in Supplementary Table S2.

### 2.28 Transcriptome sequencing and analysis

Transcriptome sequencing included two groups: control (CON) and HAMA-Exo-MnO_2_ (HEM). Total RNA was extracted using TRIzol, stored at −80 °C, and submitted to Majorbio Co., Ltd. for sequencing and bioinformatics analysis using their cloud platform.

### 2.29 Validation of transcriptomic findings

Based on transcriptomic results focusing on oxidative stress recovery, chondrocytes were challenged with H_2_O_2_ (50 μM), followed by *RT-qPCR*, immunofluorescence, and Western blot to validate key differentially expressed markers. Mitochondrial membrane potential was assessed using a JC-1 kit (Beyotime, Shanghai, China).

### 2.30 Statistical analysis

Data are presented as mean ± standard deviation (SD) from at least three independent experiments (n ≥ 3). Statistical analyses were performed using GraphPad Prism 8.0 (GraphPad Software, San Diego, CA, USA). Comparisons between two groups were conducted using an unpaired two-tailed Student's t-test, and comparisons among three or more groups were performed using one-way ANOVA followed by Tukey's post hoc test. Significance was defined as ns *P* > 0.05, ∗*P* < 0.05, ∗∗*P* < 0.01, ∗∗∗*P* < 0.001, and ∗∗∗∗*P* < 0.0001.

## 3 Results

### 3.1 Preparation and characterization of exosomes

**Fig. 1:**
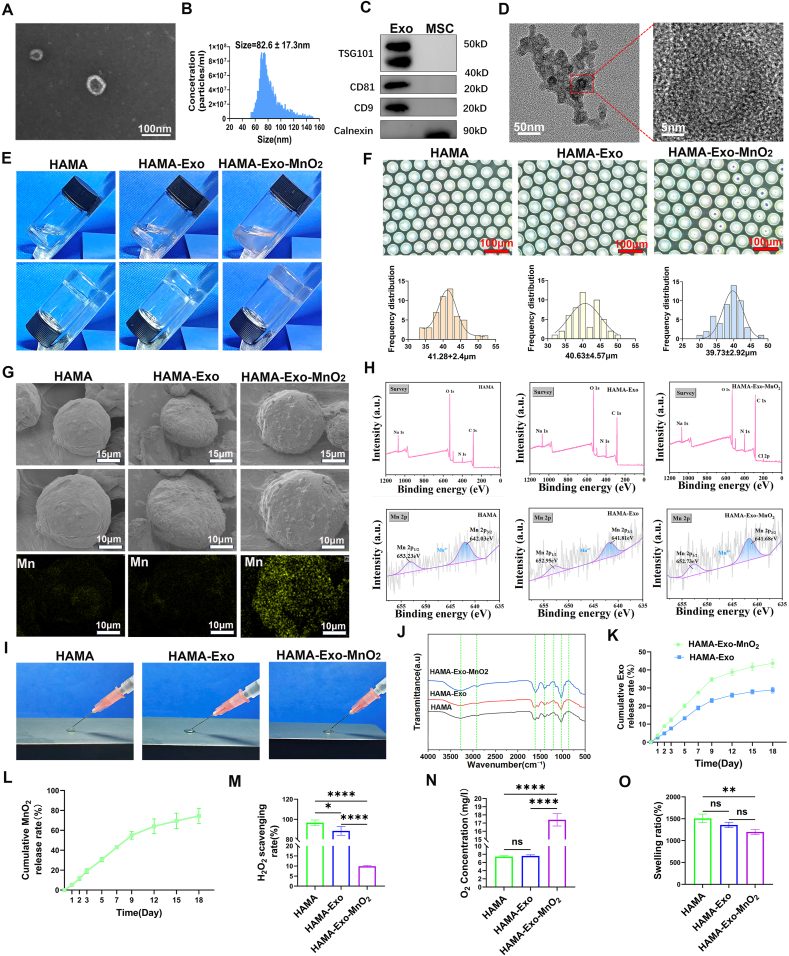
Extraction of exosomes and preparation and characterization of hydrogel microspheres. (A), (B) Transmission electron microscopy and particle size distribution of exosomes. (C) Western blot identification of specific markers of exosomes. (D) Transmission Electron Micrograph of MnO_2_. (E) Photo-crosslinking properties of hydrogel microspheres. (F) Bright field image of hydrogel microspheres and particle size distribution. (G) Scanning electron microscopy and mapping images of hydrogel microspheres. (H) XPS analysis of hydrogel microspheres. (I) Injectable properties of hydrogel microspheres. (J) Fourier transform infrared spectra of hydrogel microspheres. (K) Exosome release of hydrogel microspheres. (L) Cumulative release curve of MnO_2_. (M) Scavenging efficiency of H_2_O_2_ (n = 3). (N) Content of O_2_ generation (n = 3). (O) Swelling ratio of microspheres (n = 3). ns *P* > 0.05, ∗*P* < 0.05, ∗∗*P* < 0.01, ∗∗∗∗*P* < 0.0001.

Transmission electron microscopy (TEM) showed that the isolated vesicles displayed a typical exosome-like morphology, with diameters ranging from 55 to 150 nm and an average size of 82.6 ± 17.3 nm (Fig. 1A and B). Western blot further verified the presence of exosomal markers (TSG101, CD81, and CD9), while the endoplasmic reticulum protein calnexin was undetectable, indicating minimal cellular contamination (Fig. 1C). Collectively, these data demonstrate successful isolation of hUC-MSC–derived exosomes.

### 3.2 Morphology and phase identification of MnO_₂_ nanoparticles

TEM revealed that the MnO_2_ nanozyme exhibited a rod-like nanostructure assembled into dendrite-like architectures. High-resolution TEM (HRTEM) displayed clear lattice fringes with an interplanar spacing of ∼0.31 nm, and the corresponding diffraction pattern showed weak crystalline features, consistent with α-MnO_2_ (Fig. 1D).

### 3.3 Hydrogel crosslinking and microsphere morphology

All formulations formed stable hydrogels within 15 s under 365 nm UV irradiation (Fig. 1E), suggesting that incorporation of Exo and MnO_2_ did not compromise photocrosslinking. Optical microscopy showed that all microspheres were well-formed and spherical, with HAMA-Exo-MnO_2_ exhibiting a slightly larger diameter than HAMA (Fig. 1F). Scanning electron microscopy (SEM) revealed smooth surfaces for HAMA and HAMA-Exo microspheres, whereas HAMA-Exo-MnO_2_ microspheres displayed visible irregular pores. Consistently, energy-dispersive X-ray spectroscopy (EDS) mapping demonstrated a markedly higher Mn signal on the surface of HAMA-Exo-MnO_2_ compared with controls, confirming Mn incorporation (Fig. 1G).

### 3.4 Composition analysis

XPS survey spectra of all groups displayed C 1s, O 1s, Na 1s, and N 1s peaks, attributable to the HAMA matrix and proteinaceous components from exosomes. Mn 2p signals (640–655 eV) were detected exclusively in the HAMA-Exo-MnO_2_ group, confirming MnO_2_ loading. High-resolution Mn 2p spectra showed Mn 2p_3_/_2_ at 641.68 eV and Mn 2p_1_/_2_ at 652.73 eV, with a spin–orbit splitting of 11.05 eV, consistent with Mn^4+^ in MnO_2_ and indicating no apparent change in Mn valence during incorporation (Fig. 1H).

FTIR spectra of HAMA, HAMA-Exo, and HAMA-Exo-MnO_2_ showed the characteristic bands of the HAMA matrix, including O–H stretching (3500–3200 cm^−1^), C–H stretching (3000–2800 cm^−1^), C=O stretching (1720–1650 cm^−1^), and amide bands (1600–1550 cm^−1^), indicating that the polymer backbone remained intact after cargo loading. Compared with HAMA, HAMA-Exo exhibited only a slight enhancement in the amide region, suggesting that exosomes were mainly incorporated via physical encapsulation without altering the chemical structure of HAMA. Notably, HAMA-Exo-MnO_2_ showed weakened C–H/C=O band intensities and broader absorption in the low-wavenumber region, consistent with MnO_2_ incorporation and its interactions with the polymer network (Fig. 1J).

### 3.5 Injectability

The injectability evaluation showed that suspensions of HAMA, HAMA-Exo, and HAMA-Exo-MnO_2_ microspheres in PBS could be smoothly extruded through a 25 G needle, without clogging, demonstrating good injectability (Fig. 1 I).

### 3.6 Sustained-release properties of microspheres

In vitro sustained-release profiles revealed distinct cumulative release patterns of exosomes from HAMA-Exo and HAMA-Exo-MnO_2_ microspheres (Fig. 1K). The HAMA-Exo group demonstrated a relatively slow and sustained release, gradually increasing toward a plateau. In contrast, the HAMA-Exo-MnO_2_ group exhibited accelerated release kinetics, showing a notably faster initial release trend before stabilizing at a higher final cumulative release. In both groups, the release remained incomplete, suggesting that a proportion of exosomes were retained within the microsphere network. Furthermore, the cumulative release of Mn from the HAMA-Exo-MnO_2_ microspheres (Fig. 1L) displayed a sustained progression over the 18-day observation period. The curve exhibited a relatively steady release rate during the initial phase, followed by a gradual deceleration, demonstrating a sustained-release behavior within this timeframe.

### 3.7 Catalytic activity and H_2_O_2_ scavenging efficiency

As shown in Fig. 1M, the residual H_2_O_2_ levels in the HAMA-Exo-MnO_2_ group were significantly lower than those in the HAMA and HAMA-Exo groups. Correspondingly, the O_2_ concentration in the HAMA-Exo-MnO_2_ group was notably higher than that of the other two groups (Fig. 1N). These results indicate that the incorporation of MnO_2_ nanoparticles endows the composite microspheres with the catalytic capacity to decompose H_2_O_2_ and generate O_2_.

### 3.8 Swelling ratio

As shown in Fig. 1O, all microspheres exhibited excellent swelling capacity. The sequential incorporation of exosomes and MnO_2_ nanoparticles resulted in a slight and gradual decrease in the swelling ratio compared to the pure HAMA group. Despite this minor reduction, the composite microspheres still maintained robust water retention, ensuring adequate structural stability for sustained release in the joint cavity.

### 3.9 Chondrocyte isolation and exosome uptake

**Fig. 2:**
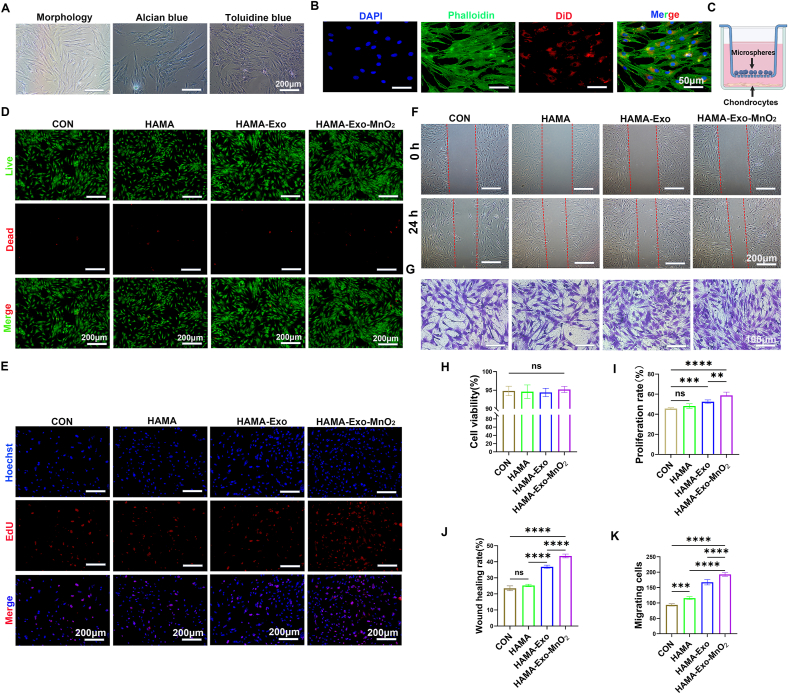
Extraction of chondrocytes and biocompatibility evaluation of microspheres. (A) Extraction and identification of chondrocytes by specific staining. (B) Uptake of exosomes by chondrocytes. (C) Scheme of chondrocytes co-culture with hydrogel microspheres (Created with BioRender.com). (D) Live/dead staining of chondrocytes co-cultured with hydrogel microspheres. (E) EdU staining of chondrocytes co-cultured with hydrogel microspheres. (F) Wound healing assay of chondrocytes co-cultured with hydrogel microspheres. (G) Transwell assay of chondrocytes co-cultured with hydrogel microspheres. (H) Quantitative analysis of Live/Dead staining. (I) Quantitative analysis of EdU staining. (J) Quantitative Analysis of Wound healing assay. (K) Quantitative analysis of transwell assay. ns *P* > 0.05, ∗∗*P* < 0.01, ∗∗∗*P* < 0.001, ∗∗∗∗*P* < 0.0001.

Primary human chondrocytes were isolated from articular cartilage and exhibited typical morphology under light microscopy, forming a dense monolayer with spindle-shaped to polygonal cells. Alcian blue staining showed strong positive signals, indicating abundant glycosaminoglycan (GAG) deposition in the extracellular matrix. Toluidine blue staining further revealed metachromatic purple regions, consistent with a proteoglycan-rich matrix and a maintained chondrocytic phenotype (Fig. 2A).

To evaluate cellular internalization, DiD-labeled exosomes were incubated with chondrocytes. Fluorescence imaging demonstrated prominent perinuclear DiD signals within the cytoplasm after 12 h, indicating efficient uptake of exosomes by chondrocytes (Fig. 2B).

### 3.10 Biocompatibility evaluation of microspheres

The cytocompatibility of microspheres was assessed using a Transwell co-culture system. Live/Dead staining showed that chondrocytes maintained high viability after 72 h of co-culture with microspheres across all groups (Fig. 2D), with quantitative viability exceeding 95% (Fig. 2H).

EdU staining revealed a higher fraction of proliferating cells in the HAMA-Exo-MnO_2_ group compared with the control (Fig. 2E and I), indicating enhanced proliferation. Consistently, wound healing assay showed faster wound closure in the HAMA-Exo-MnO_2_ group (Fig. 2F and J). Transwell migration assays further demonstrated an increased number of migrated chondrocytes upon exposure to HAMA-Exo-MnO_2_ microspheres (Fig. 2G and K). Together, these results suggest that HAMA-Exo-MnO_2_ microspheres are cytocompatible and can promote chondrocyte proliferation and migration in vitro.

### 3.11 Microspheres alleviate OA-like pathological changes in chondrocytes

**Fig. 3:**
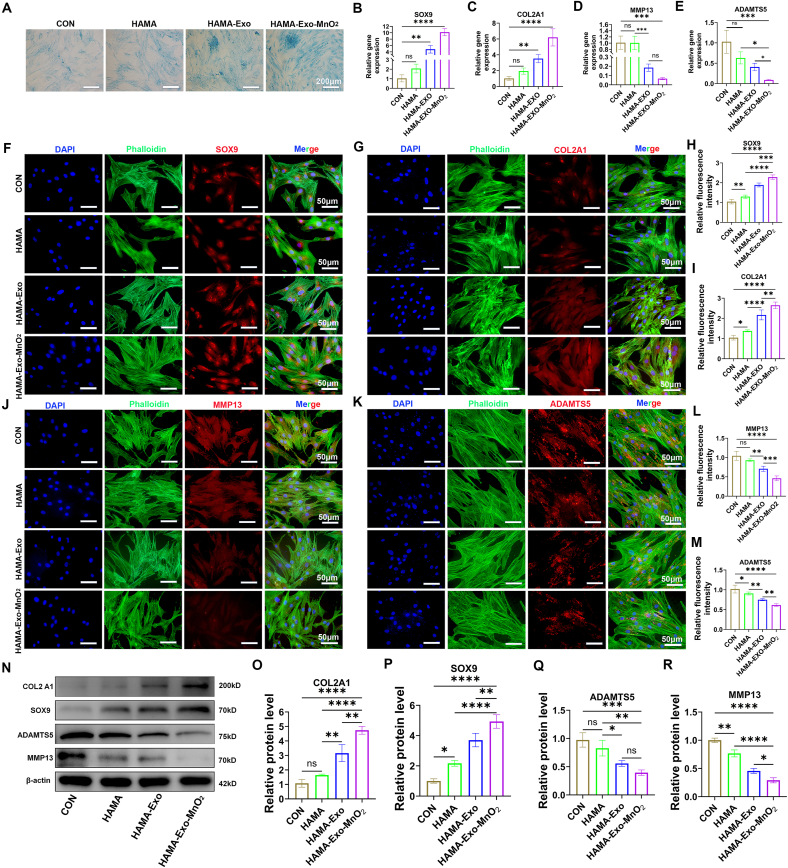
HAMA-Exo-MnO_2_ hydrogel microspheres alleviate pathological progression of OA chondrocytes. (A) Alcian blue staining of chondrocytes co-cultured with hydrogel microspheres. (B), (C), (D), (E) *RT-qPCR* of chondrocytes co-cultured with hydrogel microspheres. (F), (G), (J), (K) Immunofluorescence staining of chondrocytes co-cultured with hydrogel microspheres. (H), (I), (L), (M) Quantitative analysis of immunofluorescence of chondrocytes co-cultured with hydrogel microspheres. (N) Western blot of chondrocytes co-cultured with hydrogel microspheres. (O), (P), (Q), (R) Quantitative analysis of Western blot. ns *P* > 0.05, ∗*P* < 0.05, ∗∗*P* < 0.01, ∗∗∗*P* < 0.001, ∗∗∗∗*P* < 0.0001.

Alcian blue staining showed enhanced acidic mucopolysaccharide deposition in OA-like chondrocytes treated with HAMA-Exo-MnO_2_ microspheres, suggesting improved matrix production compared with the control conditions (Fig. 3A). Consistently, *RT-qPCR* demonstrated that HAMA-Exo-MnO_2_ upregulated anabolic/chondrogenic markers (COL2A1 and SOX9) while downregulating catabolic markers (MMP13 and ADAMTS5) in OA-like chondrocytes (Fig. 3B–E).

Immunofluorescence analysis further corroborated these transcriptional changes: COL2A1 and SOX9 fluorescence intensities were increased in the HAMA-Exo-MnO_2_ group (Fig. 3F–I), whereas MMP13 and ADAMTS5 signals were reduced (Fig. 3J–M). Western blot yielded comparable trends at the protein level, showing elevated COL2A1 and SOX9 and decreased MMP13 and ADAMTS5 in chondrocytes exposed to HAMA-Exo-MnO_2_ microspheres (Fig. 3N–R). Together, these results indicate that HAMA-Exo-MnO_2_ microspheres shift OA-like chondrocytes toward an anabolic phenotype and suppress matrix-degrading programs in vitro.

### 3.12 Antioxidant properties of hydrogel microspheres

**Fig. 4:**
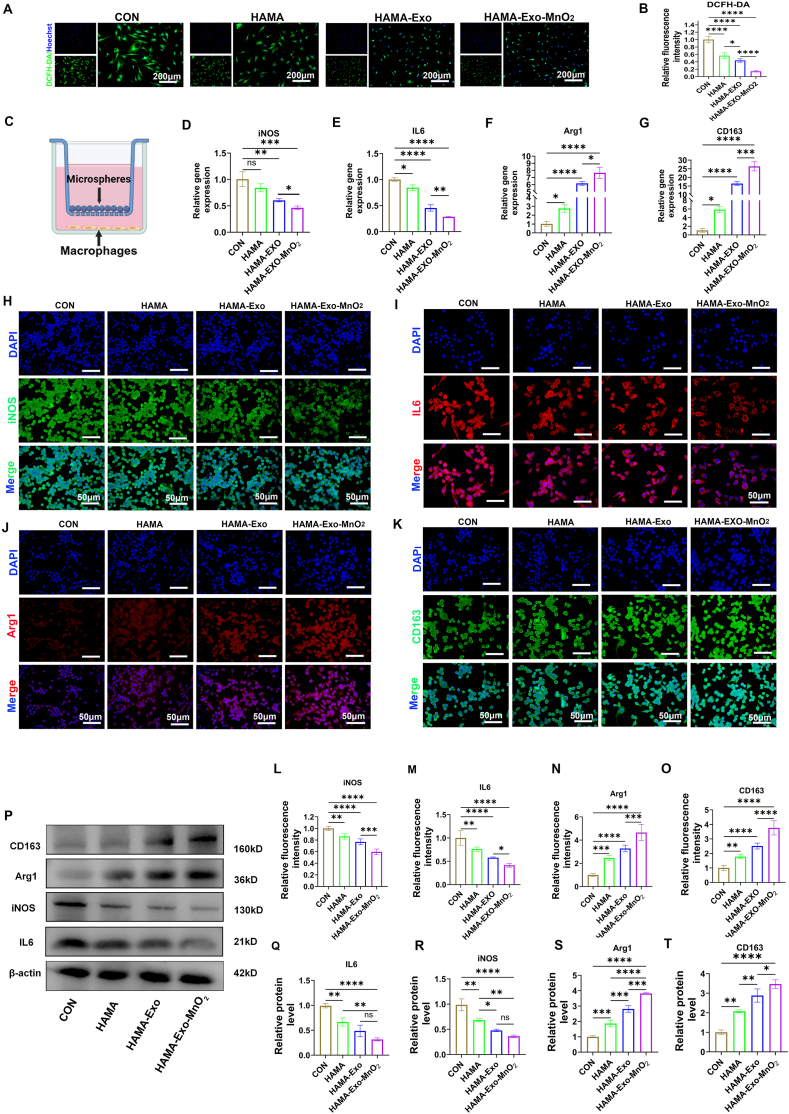
HAMA-Exo-MnO_2_ hydrogel microspheres scavenge ROS and regulate inflammatory microenvironment. (A) Fluorescence images of ROS scavenging by hydrogel microspheres. (B) Quantitative analysis of ROS scavenging effect of hydrogel microspheres on H₂O₂-challenged chondrocytes. (C) Scheme of hydrogel microspheres co-cultured with RAW 264.7 (Created with BioRender.com). (D-G) *RT-qPCR* of RAW 264.7 co-cultured with hydrogel microspheres. (H-K) Immunofluorescence staining of RAW 264.7 co-cultured with hydrogel microspheres. (L-O) Quantitative analysis of immunofluorescence of RAW 264.7 co-cultured with hydrogel microspheres. (P) Western blot of RAW 264.7 co-cultured with hydrogel microspheres. (Q-T) Quantitative analysis of Western blot. ns *P* > 0.05, ∗*P* < 0.05, ∗∗*P* < 0.01, ∗∗∗*P* < 0.001, ∗∗∗∗*P* < 0.0001.

As depicted by the DCFH-DA fluorescence staining (Fig. 4A), the H_2_O_2_-challenged control group exhibited a prominent green fluorescent signal, indicating an accumulation of intracellular ROS. Following treatment, the fluorescence intensity progressively diminished across the HAMA, HAMA-Exo, and HAMA-Exo-MnO_2_ groups. Quantitative analysis (Fig. 4B) further verified this stepwise reduction. Consistently, these findings demonstrated that the HAMA-Exo-MnO_2_ microspheres reduced intracellular ROS levels more effectively than the other groups, thereby supporting their enhanced ROS-scavenging capacity.

### 3.13 Anti-inflammatory effects via macrophage polarization

Macrophage polarization was assessed by measuring M1- and M2-associated markers. *RT-qPCR* showed that HAMA-Exo-MnO_2_ microspheres downregulated the M1-related genes iNOS and IL-6, while upregulating the M2-related genes Arg1 and CD163 (Fig. 4D–G). Immunofluorescence further confirmed reduced iNOS/IL-6 signals and enhanced Arg1/CD163 signals in the HAMA-Exo-MnO_2_ group (Fig. 4H–O). Western blot yielded consistent trends at the protein level, showing decreased iNOS and IL-6 and increased Arg1 and CD163 after treatment with HAMA-Exo-MnO_2_ microspheres (Fig. 4P–T). Collectively, these results suggest that HAMA-Exo-MnO_2_ microspheres suppress pro-inflammatory M1 polarization while promoting an anti-inflammatory M2 phenotype.

### 3.14 Sustained retention effect of exosomes in joint cavity

*In vivo* fluorescence imaging (Fig. S3A) revealed a rapid signal decay for free exosomes over 12 days, with abdominal fluorescence emerging by day 3 likely due to rapid systemic clearance via the synovium. Conversely, signals from the HAMA-Exo and HAMA-Exo-MnO_2_ groups persisted significantly longer. The slightly faster attenuation in the HAMA-Exo-MnO_2_ group relative to the HAMA-Exo group aligned well with its accelerated in vitro release kinetics. Quantitative analysis (Fig. S3B) further confirmed that microsphere encapsulation markedly mitigated signal loss, effectively prolonging the intra-articular retention of exosomes.

### 3.15 Knee function assessment

**Fig. 5:**
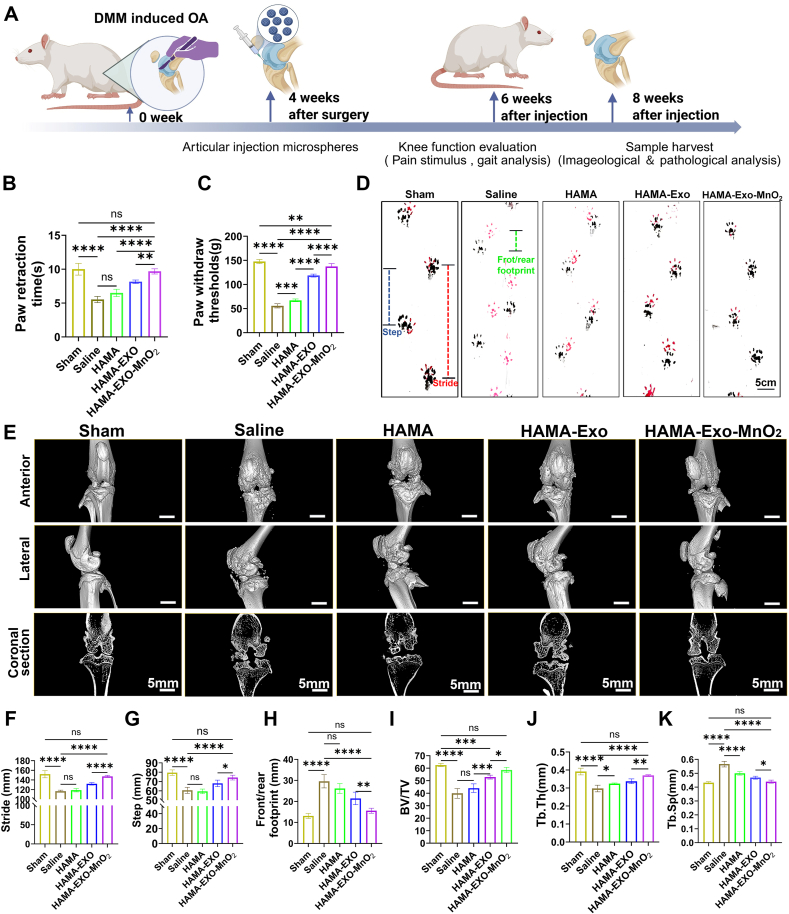
HAMA-Exo-MnO_2_ hydrogel microspheres restored knee joint function and reduced pathological osteophyte proliferation in OA SD rats. (A) Scheme of animal experiments(Created with BioRender.com). (B) Hot plate test after 6 weeks of hydrogel microspheres treatment. (C) Mechanical withdrawal threshold after 6 weeks of hydrogel microspheres treatment. (D) Gait images after 6 weeks of hydrogel microspheres treatment. (E) Radiological images after 8 weeks of hydrogel microsphere treatment. (F), (G), (H) Quantitative analysis of gait images. (I), (J), (K) Quantitative analysis of radiological images. ns *P* > 0.05, ∗*P* < 0.05, ∗∗*P* < 0.01, ∗∗∗*P* < 0.001, ∗∗∗∗*P* < 0.0001.

Behavioral testing (Fig. S5) indicated that intra-articular administration of HAMA-Exo-MnO_2_ microspheres alleviated pain-related behaviors and improved knee function in DMM-induced OA rats. In the hot-plate test, rats receiving HAMA-Exo-MnO_2_ exhibited a longer withdrawal latency, suggesting reduced thermal hyperalgesia; however, the latency remained slightly lower than that of the sham group (Fig. 5B). In the mechanical nociception assay, the HAMA-Exo-MnO_2_ group showed a significantly higher withdrawal threshold than the saline-treated OA group and approached the sham level (Fig. 5C).

Gait analysis further supported functional recovery. Compared with the saline-treated OA rats, the HAMA-Exo-MnO_2_ group displayed significantly increased stride and step lengths, alongside a markedly reduced front/rear footprint distance, presenting a gait pattern closely resembling that of the sham animals (Fig. 5D). Notably, quantitative evaluations of these three parameters showed no significant differences between the HAMA-Exo-MnO_2_ and sham groups (Fig. 5F–H), indicating an effective restoration of normal motor coordination. Collectively, these results suggest that HAMA-Exo-MnO_2_ microspheres partially restore joint function and reduce pain-related behaviors following DMM surgery.

### 3.16 Radiographic evaluation

Representative X-ray radiographs revealed pronounced joint space narrowing and extensive osteophyte formation in the saline and HAMA groups compared with the sham group. While HAMA-Exo treatment provided moderate structural alleviation, the HAMA-Exo-MnO_2_ group achieved superior preservation of joint integrity, with radiographic features resembling those observed in the sham group (Fig. S6). Micro-CT analysis showed evident osteophyte formation in OA joints compared with the sham group, with the saline group displaying the most pronounced changes (Fig. 5E). Quantitative micro-CT assessment of periarticular bone parameters revealed decreased BV/TV and Tb.Th and increased Tb.Sp in the saline and HAMA groups, indicating substantial subchondral bone deterioration after DMM. In contrast, rats treated with HAMA-Exo and HAMA-Exo-MnO_2_ exhibited partial recovery of these parameters, with the HAMA-Exo-MnO_2_ group showing values closer to those of the sham group (Fig. 5I–K). These data indicate that HAMA-Exo-MnO_2_ treatment mitigates micro-CT–detectable OA-associated structural changes in periarticular bone.

### 3.17 Histopathological examination

**Fig. 6:**
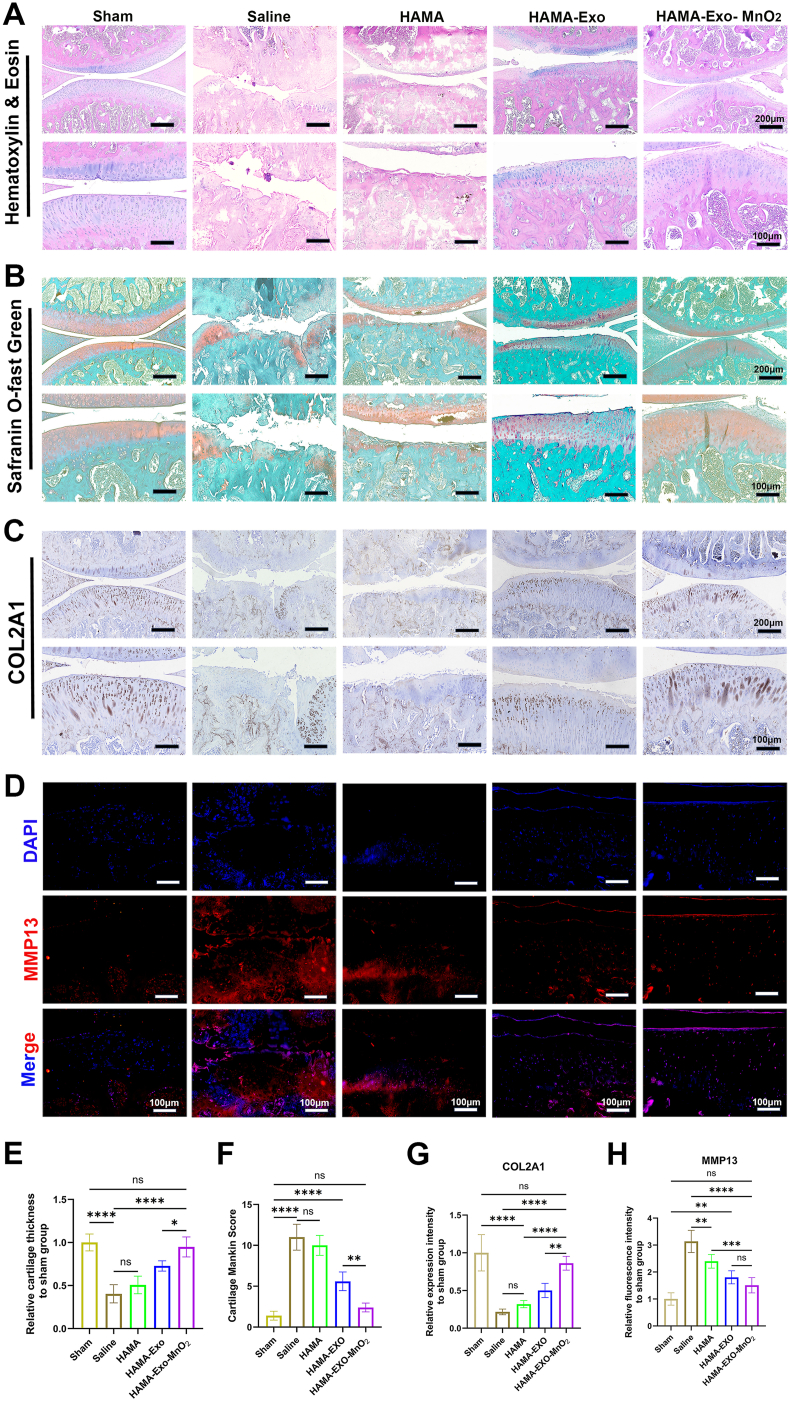
HAMA-Exo-MnO_2_ hydrogel microspheres alleviate articular cartilage degeneration in OA SD rats. (A) Representative image of H&E stain. (B) Representative image of Safranin-O-Fast Green stain. (C) Representative image of immunohistochemical staining of COL2A1. (D) Representative image of immunofluorescence staining of MMP13. (E) Quantitative analysis of cartilage relative thickness. (F) Cartilage Mankin score. (G) Quantitative analysis of COL2A1. (H) Quantitative analysis of MMP13. ns *P* > 0.05, ∗*P* < 0.05, ∗∗*P* < 0.01, ∗∗∗*P* < 0.001, ∗∗∗∗*P* < 0.0001.

Histological staining corroborated the therapeutic effects observed in behavioral and micro-CT analyses. H&E staining showed intact cartilage architecture in the Sham group, characterized by a smooth articular surface, preserved matrix integrity, and orderly chondrocyte distribution. In contrast, severe cartilage degeneration was observed in the Saline and HAMA groups, including surface fibrillation/erosion, matrix disorganization and thinning, chondrocyte loss, and focal exposure of subchondral bone. The HAMA-Exo group displayed partial protection, with reduced cartilage destruction but residual surface fissures and matrix depletion. Notably, the HAMA-Exo-MnO_2_ group exhibited a markedly improved cartilage morphology, with a smoother surface and more preserved tissue structure, approaching the Sham phenotype (Fig. 6A).

Safranin O/Fast Green staining further demonstrated strong and homogeneous proteoglycan staining with a clear tidemark in the sham group, whereas the saline and HAMA groups showed pronounced loss of Safranin O intensity and disrupted cartilage layering. The HAMA-Exo group showed moderate retention of proteoglycans, while the HAMA-Exo-MnO_2_ group exhibited the greatest preservation of Safranin O staining among OA groups, consistent with improved extracellular matrix integrity (Fig. 6B).

Immunohistochemical staining of COL2A1 (Fig. 6C–G) revealed robust and homogeneous staining throughout the cartilage matrix in the Sham group. In contrast, the Saline group exhibited markedly diminished COL2A1 expression, indicating substantial loss of type II collagen. Partial restoration of COL2A1 was observed in the HAMA and HAMA-Exo groups, while the HAMA-Exo-MnO_2_ group displayed the most intense and widespread staining, closely resembling Sham levels. Correspondingly, immunofluorescence for MMP13 (Fig. 6D–H) showed pronounced red fluorescence in the Saline group, reflecting elevated matrix catabolism. This signal was moderately attenuated in the HAMA and HAMA-Exo groups and was substantially suppressed in the HAMA-Exo-MnO_2_ group, indicating effective inhibition of degradative activity. Quantitative assessment of cartilage thickness (Fig. 6E) demonstrated significant thinning in the Saline group compared to Sham, with partial recovery in the HAMA and HAMA-Exo groups. Notably, the HAMA-Exo-MnO_2_ group exhibited the greatest thickness, approaching Sham levels. Histopathological evaluation via the Mankin score (Fig. 6F) further confirmed severe cartilage degeneration in the Saline group. Scores were progressively lower in the HAMA and HAMA-Exo groups, with the HAMA-Exo-MnO_2_ group achieving the lowest score, showing no significant difference from the Sham group.

### 3.18 Transcriptome-guided mechanistic insights into HAMA-Exo-MnO_2_ treatment

**Fig. 7:**
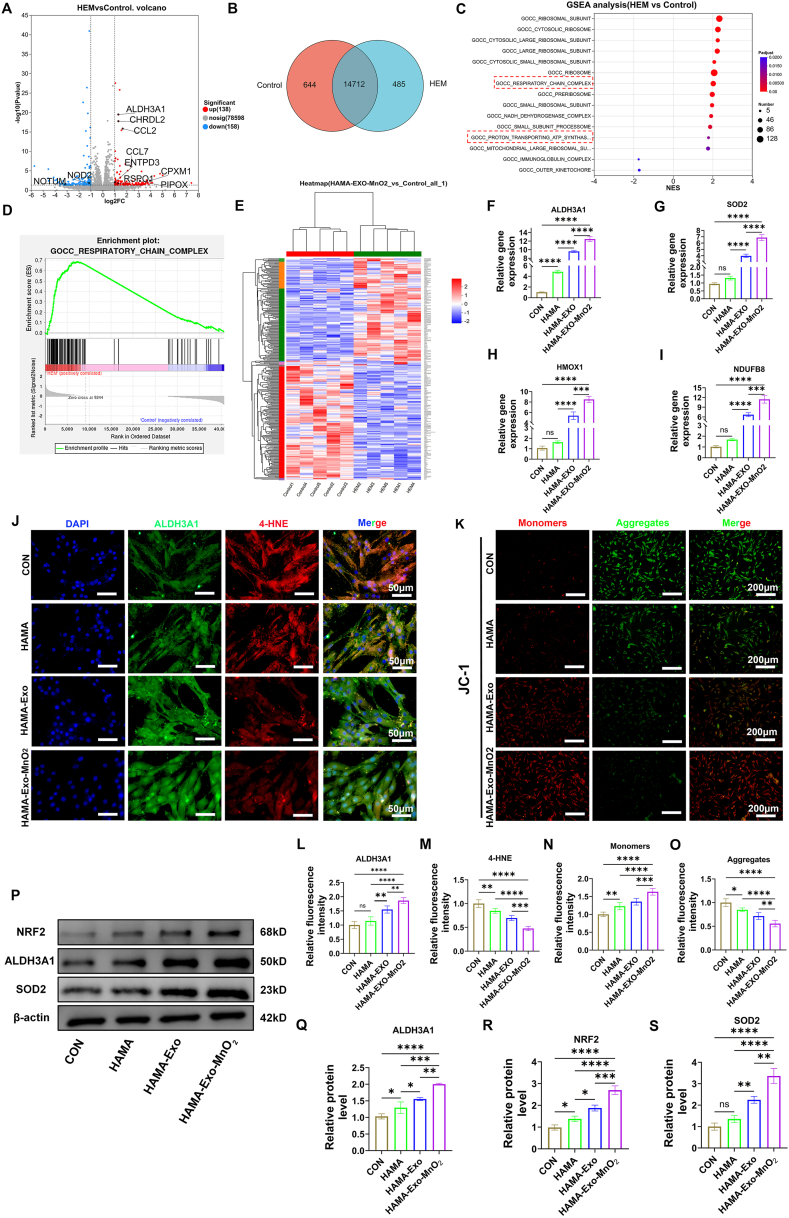
Potential mechanism of HAMA-Exo-MnO_2_ hydrogel microspheres in the treatment of osteoarthritis. (A) The volcano plot showing the DEGs of HAMA-Exo-MnO_2_ vs. control. (B) Venn diagram showing the overlap and unique expressed genes between HAMA-Exo-MnO_2_ and Control groups. (C) GSEA bubble plot showing enriched pathways in the HAMA-Exo-MnO_2_ group. (D) Representative GSEA enrichment plot for the GO term “GOCC_RESPIRATORY_CHAIN_COMPLEX,” demonstrating positive enrichment in the HAMA-Exo-MnO_2_ group. (E) Hierarchical clustering heatmap of DEGs, with samples clearly separated into Control and HAMA-Exo-MnO_2_ clusters (red: upregulated, blue: downregulated). (F), (G), (H), (I) *RT-qPCR* validation of ALDH3A1, SOD2, HMOX1, NDUFB8. (J) Representative immunofluorescence images of ALDH3A1 and 4-HNE. (K) Representative JC-1 staining images showing mitochondrial membrane potential. (L) Quantitative analysis of ALDH3A1 immunofluorescence intensity. (M)Quantitative analysis of 4-HNE immunofluorescence intensity. (N) Quantitative analysis of JC-1 monomers. (O) Quantitative analysis of JC-1 aggregates. (P) Western blot analysis of NRF2, ALDH3A1, and SOD2. (Q), (R), (S) Quantitative analysis of Western blot. ns *P* > 0.05, ∗*P* < 0.05, ∗∗*P* < 0.01, ∗∗∗*P* < 0.001, ∗∗∗∗*P* < 0.0001.

To gain mechanistic insight into how HAMA-Exo-MnO_2_ microspheres modulate OA chondrocyte phenotypes, RNA sequencing was performed on human OA chondrocytes co-cultured with HAMA-Exo-MnO_2_ (HEM) or control conditions. Differential expression analysis revealed a distinct transcriptional signature in the HEM group, with 138 genes significantly upregulated and 158 genes significantly downregulated. Notably, the antioxidant-associated gene ALDH3A1 was among the most prominently upregulated transcripts, whereas inflammatory-related genes such as NOD2 exhibited a downregulation trend (Fig. 7A). Venn analysis identified both shared and condition-specific transcripts, indicating that HEM induced distinct gene expression changes relative to the control group (Fig. 7B). Gene set enrichment analysis (GSEA) further indicated that HEM treatment was associated with activation of pathways related to mitochondrial function and protein synthesis. Among the enriched gene sets, oxidative phosphorylation and respiratory chain related terms (e.g., GOBP_RESPIRATORY_CHAIN_COMPLEX, and GOBP_MITOCHONDRIAL_ELECTRON_TRANSPORT_NADH_TO_UBIQUINONE) showed the strongest positive enrichment in the HEM group, suggesting enhanced transcriptional programs supporting mitochondrial electron transport and energy metabolism (Fig. 7C). A representative enrichment plot for GOCC_RESPIRATORY_CHAIN_COMPLEX further illustrated significant positive enrichment in the HEM group (Fig. 7D). Hierarchical clustering demonstrated clear separation between HEM-treated samples and controls, consistent with robust treatment-associated transcriptional remodeling (Fig. 7 E).

Based on the transcriptomic findings, we focused on the antioxidant recovery response centered on ALDH3A1 and NRF2-related signaling. *RT-qPCR* confirmed that HEM treatment upregulated ALDH3A1, concomitantly increased SOD2 (a key NRF2-responsive antioxidant gene), and elevated NDUFB8, a marker associated with mitochondrial respiratory chain function (Fig. 7F–I). Immunofluorescence staining further showed enhanced ALDH3A1 signals accompanied by reduced 4-HNE staining, indicating attenuation of lipid peroxidation (Fig. 7J, L and M). Consistently, JC-1 assays demonstrated restoration of mitochondrial membrane potential (ΔΨm) in HEM-treated chondrocytes under oxidative challenge, supporting improved mitochondrial homeostasis (Fig. 7K, N and O). At the protein level, Western blot confirmed increased ALDH3A1 expression along with upregulation of NRF2 and SOD2 (Fig. 7P–S). Together, these multi-level data suggest that HAMA-Exo-MnO_2_ microspheres activate an ALDH3A1/NRF2-linked antioxidant program, reduce lipid peroxidation, and promote mitochondrial functional recovery in OA chondrocytes.

## 4 Discussion

Osteoarthritis (OA) is a multifactorial degenerative joint disease driven by cartilage matrix loss, persistent inflammation, and oxidative stress, which together form self-reinforcing pathogenic loops [37]. Oxidative stress–induced mitochondrial dysfunction represents a key nexus in OA progression. Excess ROS can directly damage chondrocyte DNA, proteins, and lipids and disrupt the mitochondrial electron transport chain (ETC), thereby reducing oxidative phosphorylation (OxPhos) efficiency and ATP production while further amplifying ROS generation—a vicious cycle that accelerates chondrocyte dysfunction and matrix breakdown [[38], [39], [40]]. In parallel, sustained M1-like macrophage polarization in the OA microenvironment promotes pro-inflammatory cytokine release and the induction of matrix-degrading enzymes (e.g., MMP13 and ADAMTS5), further constraining cartilage repair capacity [41,42]. Current treatments such as nonsteroidal anti-inflammatory drugs and hyaluronic acid injections largely provide symptomatic relief but typically fail to concurrently address ROS overload, mitochondrial impairment, immune dysregulation, and insufficient regeneration [43]. Here, we developed a locally retained multifunctional hydrogel microsphere system (HAMA-Exo-MnO_2_; HEM) that integrates MnO_2_ nanozyme–mediated ROS decomposition with exosome-based bioactive signaling, thereby enabling coordinated antioxidant, immunomodulatory, and pro-regenerative effects. In the DMM model, intra-articular HEM administration alleviated OA-associated pain behaviors and improved joint function while attenuating structural deterioration, supporting the stability and biocompatibility of the platform in the joint environment.

At the materials level, microspheres in all groups exhibited well-maintained spherical morphology indicating the excellent gel-forming and structural stability of the methacrylated hyaluronic acid backbone [44]. SEM revealed a more porous surface on HEM compared with the relatively smooth surfaces of HAMA and HAMA-Exo microspheres, suggesting that the incorporation of MnO_2_ nanoparticles significantly modulates the polysaccharide network density [45]. This micro-porosity is consistent with the higher cumulative release of exosomes observed in HEM. Mechanistically, the HA molecular chains in HAMA possess high-density carboxyl and hydroxyl groups, which provide sites for potential non-covalent interactions with MnO_2_ [46]. However, the embedded MnO_2_ nanoparticles primarily act as physical spacers that interfere with the tight packing of the HA polymer chains during UV-induced crosslinking, yielding a relatively loosened network with enlarged pore structures [47]. This structural expansion is particularly advantageous for carbohydrate-based delivery systems, as it facilitates the diffusion of large-molecular-weight cargos, such as exosomes, while preserving the rapid gelation kinetics inherent to the methacrylated polysaccharide [48]. Elemental mapping (EDS) and chemical analyses (FTIR and XPS) jointly confirmed successful MnO_2_ incorporation into the HAMA matrix. Notably, due to the low loading of MnO_2_ and the overwhelming signal from the HA polysaccharide backbone, FTIR and XPS characterizations revealed only minimal changes in HAMA-Exo-MnO_2_ compared with other groups, whereas EDS confirmed a distinct Mn signal. To avoid potential cytotoxicity and maintain the innate biological activity of the hyaluronic acid, higher MnO₂ loadings were not employed [49,50].

Functionally, HEM demonstrated favorable cytocompatibility in vitro and supported cellular behaviors associated with cartilage repair [51]. Exosome uptake assays showed efficient internalization by chondrocytes, with prominent perinuclear localization, consistent with cellular uptake of vesicular cargo [52]. In OA-like chondrocytes, HEM increased anabolic/chondrogenic markers (COL2A1 and SOX9) and reduced catabolic mediators (MMP13 and ADAMTS5), indicating a shift toward a matrix-preserving phenotype [53]. Beyond direct effects on chondrocytes, the immunomodulatory role of HEM is highly relevant. Persistent M1 polarization contributes significantly to chronic OA inflammation [34,35], whereas M2 polarization is associated with resolution programs and the secretion of reparative factors [26,27]. HEM reduced M1-associated markers (iNOS and IL-6) and increased M2-associated markers (Arg1 and CD163), suggesting macrophage reprogramming toward an anti-inflammatory phenotype [54]. This reprogramming may arise from the combined action of exosomal regulatory cargo and MnO_2_-driven reduction in oxidative stress, which together remodel the inflammatory microenvironment and support cartilage matrix restoration—forming an integrated antioxidant, anti-inflammatory, and regenerative therapeutic network [55].

Transcriptome profiling provided mechanistic insight into the antioxidant and mitochondrial protective effects of HEM. GSEA indicated positive enrichment of OxPhos- and respiratory chain–related gene sets, including proton motive force–driven ATP synthesis and mitochondrial electron transport from NADH to ubiquinone [56,57]. Among differentially expressed genes, ALDH3A1 emerged as a prominently upregulated antioxidant-associated factor. ALDH3A1 may contribute to the detoxification of lipid peroxidation aldehydes (e.g., 4-HNE), thereby relieving oxidative constraints on NRF2 signaling and facilitating activation of downstream antioxidant responses [[58], [59], [60], [61]]. Consistently, qPCR, immunostaining, and Western blot confirmed increased ALDH3A1 expression together with elevated NRF2 pathway components and antioxidant genes (e.g., SOD2 and HMOX1), accompanied by reduced 4-HNE and improved mitochondrial membrane potential. Collectively, these data support a dual protective mechanism: (i) direct ROS decomposition via MnO_2_ nanozyme activity and (ii) activation of an ALDH3A1/NRF2-linked antioxidant program, together contributing to improved mitochondrial homeostasis and matrix maintenance in OA chondrocytes [15,62].

Several limitations of this study should be acknowledged. First, because exosomes were designed as the core therapeutic agents and MnO_2_ as an adjunctive ROS-scavenging nanozyme, our in vivo models prioritized comparing HAMA-Exo and HAMA-Exo-MnO_2_. Although this aligns with the 3R principles and the catalytic activity of MnO_2_ was extensively validated in vitro, the absence of an independent HAMA-MnO_2_ animal group limits the ability to isolate the specific in vivo contribution of MnO₂. Second, while our analyses support NRF2 pathway activation, direct evidence of NRF2 nuclear translocation and binding remains to be established. Third, the long-term degradation, in vivo release kinetics and clearance of MnO_2_ warrant systematic evaluation to strengthen comprehensive safety assessments. Finally, translating these findings will require further validation in larger animal models, alongside detailed immunogenicity evaluations, to fully confirm the clinical potential of this delivery system.

## 5 Conclusion

In summary, we developed a locally retained hydrogel microsphere platform based on methacrylated hyaluronic acid, co-delivering MnO_2_ nanozymes and mesenchymal stem cell–derived exosomes (HAMA-Exo-MnO_2_; HEM) for multi-modal OA therapy. HEM catalyzed H_2_O_2_ decomposition and reduced oxidative stress–associated damage, as evidenced by decreased levels of the lipid peroxidation marker 4-HNE. In parallel, HEM upregulated ALDH3A1 and activated an NRF2-linked antioxidant response, increasing downstream antioxidant gene expression (e.g., HMOX1 and SOD2) and enhancing mitochondrial homeostasis, consistent with improved mitochondrial membrane potential and transcriptional enrichment of oxidative phosphorylation–related programs. Moreover, HEM promoted an anti-inflammatory macrophage phenotype (increased Arg1/CD163 and decreased iNOS/IL-6 expression), attenuated catabolic matrix degradation by downregulating MMP13 and ADAMTS5, and supported chondrogenic/anabolic markers expression by upregulating COL2A1 and SOX9, thereby favoring cartilage matrix preservation and repair. In the DMM rat model, intra-articular injection of HEM alleviated OA-associated pain behaviors, improved gait parameters, mitigated structural deterioration, and exhibited favorable compatibility. Collectively, HEM provides a multifunctional and integrated strategy that couples ROS scavenging, antioxidant pathway activation, immunomodulation, and regenerative support, highlighting its potential as a translational therapeutic approach for osteoarthritis.

## CRediT authorship contribution statement

**Jianxiang Teng:** Conceptualization, Data curation, Visualization, Writing – original draft. **Jiazhao Pan:** Data curation, Methodology, Writing – original draft. **Pengcheng Yan:** Investigation, Methodology, Writing – original draft. **Tianqi Zhou:** Investigation, Methodology. **Zihao Zou:** Software, Validation. **Xiaolin Shu:** Visualization. **Hao Liu:** Resources. **Zhuoran Zhang:** Methodology. **Sheng Zhou:** Visualization. **Qiuhan Chen:** Visualization. **Dandan Shen:** Methodology. **Xing Zhao:** Resources. **Xiaobin Tian:** Project administration, Supervision. **Long Yang:** Funding acquisition, Supervision, Writing – review & editing.

## Declaration of competing interest

The authors declare that they have no known competing financial interests or personal relationships that could have appeared to influence the work reported in this paper.

## Data Availability

Data will be made available on request.
