# Supplementary material for: Microfluidic methacrylated hyaluronic acid microspheres incorporating MnO2 and exosomes for antioxidant defense and inflammation regulation in osteoarthritis
Source: Mater Today Bio. 2026 May 29;38:103300. doi: 10.1016/j.mtbio.2026.103300 (PMC13266140; doi:10.1016/j.mtbio.2026.103300)
Supplement: Multimedia component 1 [file mmc1.docx]

**Supplementary materials**

**Supplementary** Table. 1 qPCR Primer List

| Gene | Forward primer 5’-3’ | Reverse primer 5’-3’ |
| --- | --- | --- |
| GAPDH (human) | GCAAAGTGGAGATTGTTGCCAT | CCTTGACTGTGCCGTTGAATTT |
| COL2A1 | GCTCATCCAGGGCTCCAATGATGTAG | CGGGAGGTCTTCTGTGATCGGTA |
| SOX9 | CGTGCAGCACAAGAAAGACCA | GCAGCGCCTTGAAGATAGCAT |
| ADAMTS-5 | GCCCACCCAATGGTAAATCTTT | TGACTCCTTTTGCATCAGACTGA |
| MMP13 | AACATCCAAAAACGCCAGAC | GGAAGTTCTGGCCAAAATGA |
| GAPDH (mouse) | AGAAGGTGGTGAAGCAGGCATC | CGAAGGTGGAAGAGTGGGAGTTG |
| IL6 | CCAGAGATACAAAGAAATGATGG | ACTCCAGAAGACCAGAGGAAAT |
| ‌iNOS | TGTCGCAGCTCCCTATCTTG | CATTGGCCAGCTGCTTTTGC |
| CD163 | GTGCTGGATCTCCTGGTTGTA | GGAGCGTTAGTGACAGCAGA |
| ARG1 | CTCCAAGCCAAAGTCCTTAGAG | AGGAGCTGTCATTAGGGACATC |
| ALDH3A1 | CTCTGTGACCCCTCGATCCA | TCTTCCCCGTAGAACTCTTTCA |
| SOD2 | AAGTCATCCACCCACCTCAG | CGTGGAGAGAGCATGAAAGC |
| COL2A1 | GCTCATCCAGGGCTCCAATGATGTAG | CGGGAGGTCTTCTGTGATCGGTA |
| HMOX1 | TGTGGCAGCTGTCTCAAACCTCCA | TTGAGGCTGAGCCAGGAACAGAGT |
|  |  |  |

**Supplementary** Table 2 Antibody details and dilution ratios

| Antibody name | Manufacturer | Catalog number | Dilution ratios’ | Applications |  |
| --- | --- | --- | --- | --- | --- |
| β-actin | Proteintech | 60008-1-Ig | 1:5000 | Western Blot |  |
| COL2A1 | Proteintech | 86139-2-RR | 1;2000 | Western Blot |  |
| COL2A1 | | Proteintech | 28459-1-AP | 1;200 | IF Cell |
| COL2A1 | Proteintech | 86139-2-RR | 1;2000 | IHC |  |
| SOX9 | Huabio | ET1611-56 | 1:1000 | Western Blot |  |
| SOX9 | Huabio | ET1611-56 | 1:200 | Western Blot |  |
| ADAMTS-5 | Huabio | HA722011 | 1:2000 | Western Blot |  |
| ADAMTS-5 | Huabio | HA722011 | 1:100 | IF Cell |  |
| MMP13 | Proteintech | 18165-1-AP | 1:2000 | Western Blot |  |
| MMP13 | Proteintech | 18165-1-AP | 1:200 | IF Cell |  |
| MMP13 | Proteintech | 18165-1-AP | 1:200 | IF Tissue |  |
| IL6 | Proteintech | 21865-1-AP | 1:1000 | Western Blot |  |
| IL6 | Proteintech | 21865-1-AP | 1:400 | Western Blot |  |
| ‌iNOS | Proteintech | 22226-1-AP | 1:1000 | Western Blot |  |
| ‌iNOS | Proteintech | 22226-1-AP | 1:1000 | IF Cell |  |
| CD163 | Proteintech | 16646-1-AP | 1:1000 | Western Blot |  |
| CD163 | Proteintech | 16646-1-AP | 1:400 | IF Cell |  |
| Antibody name | Manufacturer | Catalog number | Dilution ratios’ | Applications |  |
| ARG1 | Proteintech | 16001-1-AP | 1:5000 | Western Blot |  |
| ARG1 | Proteintech | 16001-1-AP | 1:500 | IF Cell |  |
| ALDH3A1 | Proteintech | 15578-1-AP | 1:20000 | Western Blot |  |
| ALDH3A1 | Proteintech | 15578-1-AP | 1:200 | IF Cell |  |
| SOD2 | Proteintech | 24127-1-AP | 1;5000 | Western Blot |  |
| 4-HNE | Proteintech | 68538-1-Ig | 1:200 | IF Cell |  |
| NRF2 | Proteintech | 16396-1-AP | 1;2000 | Western Blot |  |
| Calnexin | Huabio | ET1611-86 | 1:2000 | Western Blot |  |
| TSG101 | Huabio | ET1701-59 | 1:2000 | Western Blot |  |
| CD81 | Huabio | HA723193 | 1:2000 | Western Blot |  |
| CD9 | Huabio | HA721533 | 1:2000 | Western Blot |  |


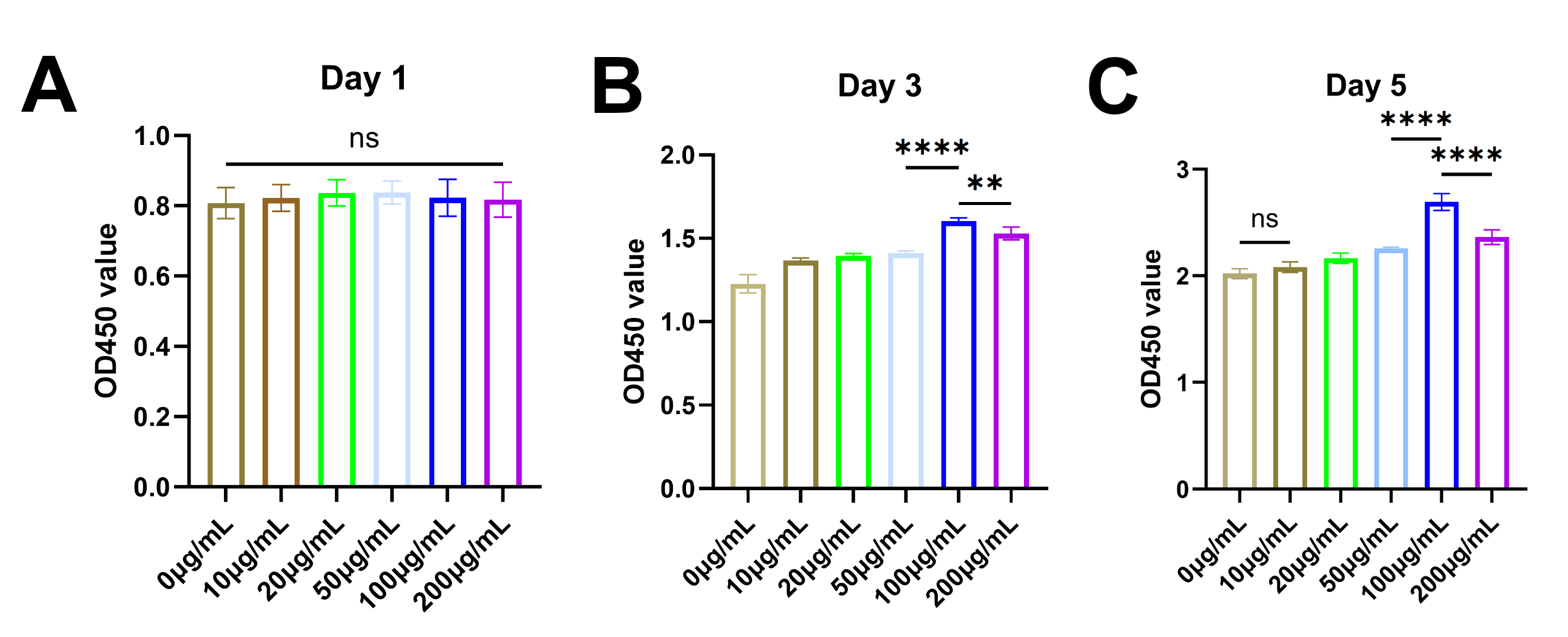


Fig. S1 Effects of Different Concentrations of MnO₂ on Chondrocytes Proliferation. (A) CCK8 of chondrocytes co-cultured with microspheres for 1 day. (B) CCK8 of chondrocytes co-cultured with microspheres for 3 day. (C) CCK8 of chondrocytes co-cultured with microspheres for 5 day.


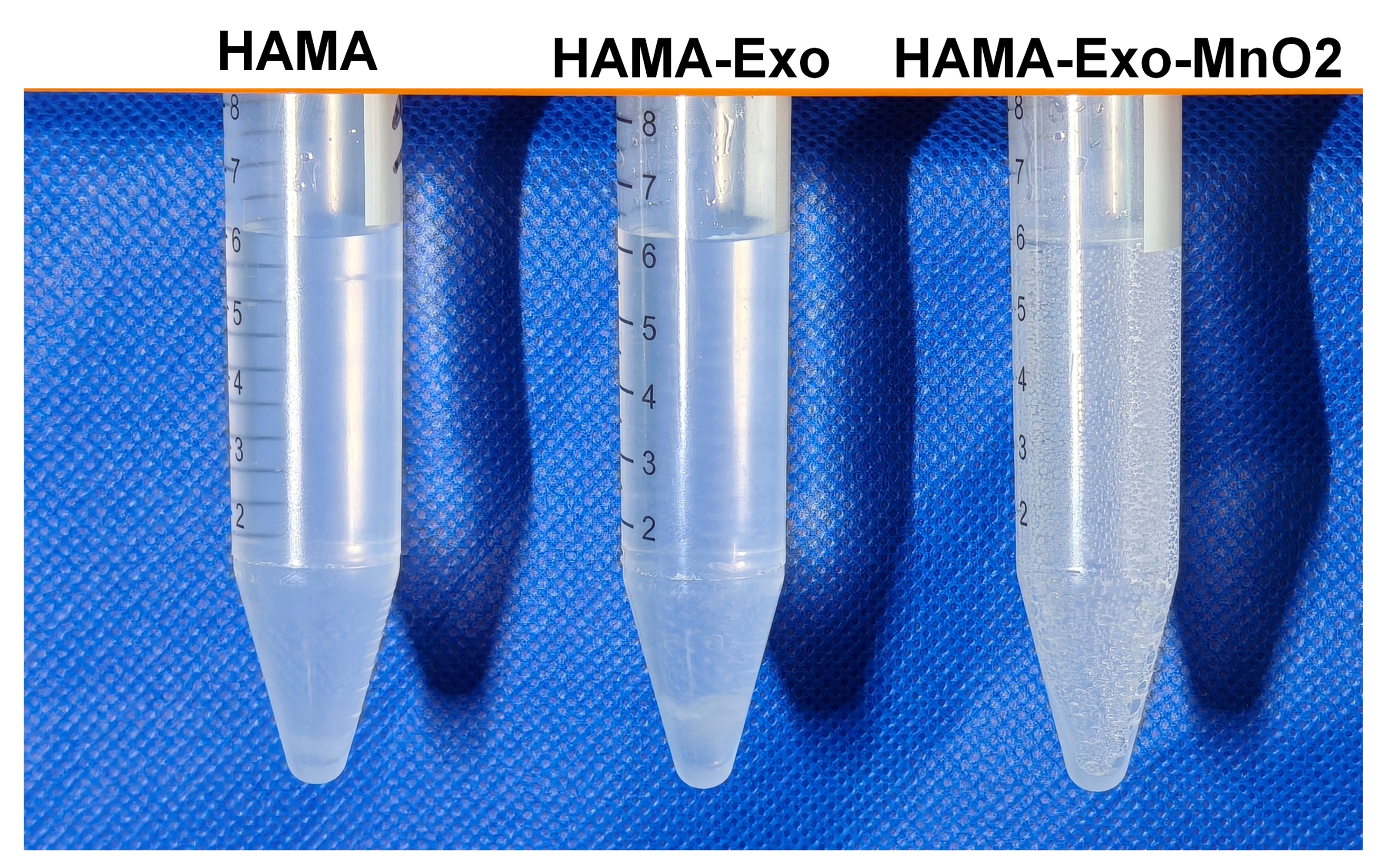


Fig. S2 Macroscopic image of microspheres incubated with H_2_O_2_ for 2 hours


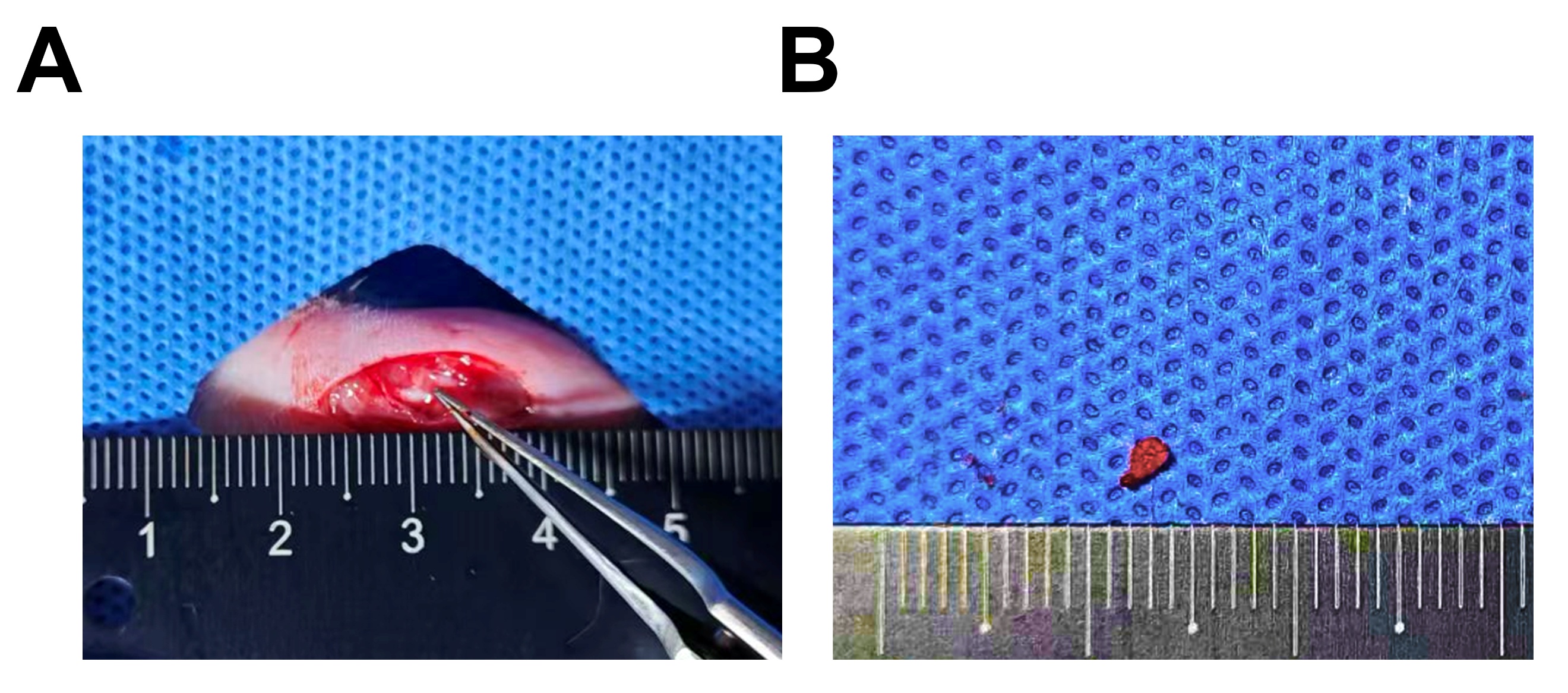


Fig. S3 Establishment of OA SD rat model by DMM surgery. (A) Exposing the medial meniscus. (B) Resected anterior horn of meniscus.


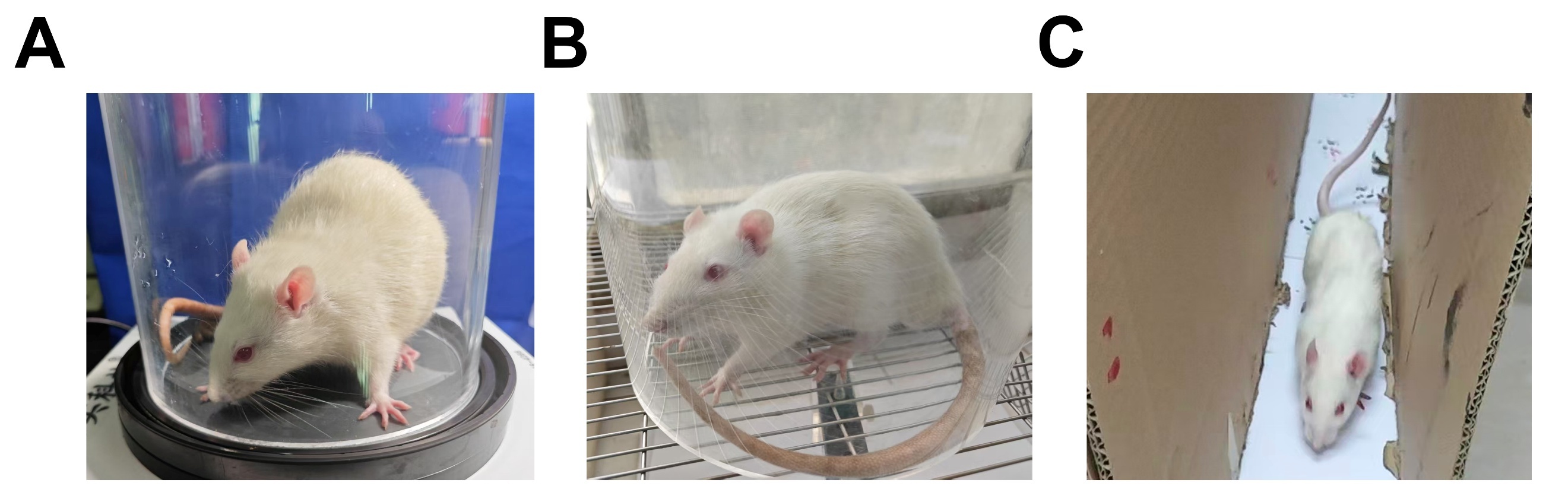


Fig. S4. Functional assessment: (A) Hot-plate test. (B) Mechanical stimulation. (C) Gait analysis.


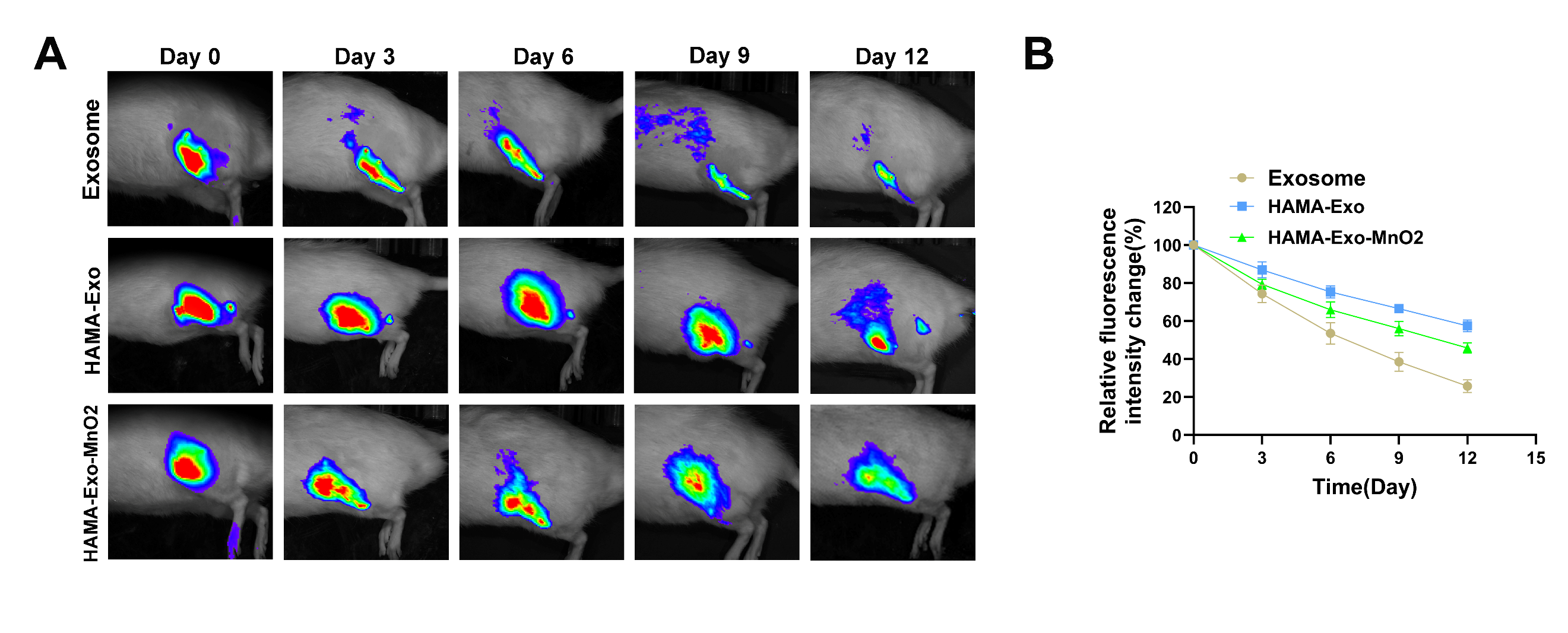


Fig. S5 In vivo fluorescence imaging of free DiD-labeled exosomes and exosome-loaded microspheres in rat knee joints. (A) Representative fluorescence images of different groups at day 0, 3, 6, 9, and 12 post-injection. (B) Relative fluorescence intensity changes over 12 days.


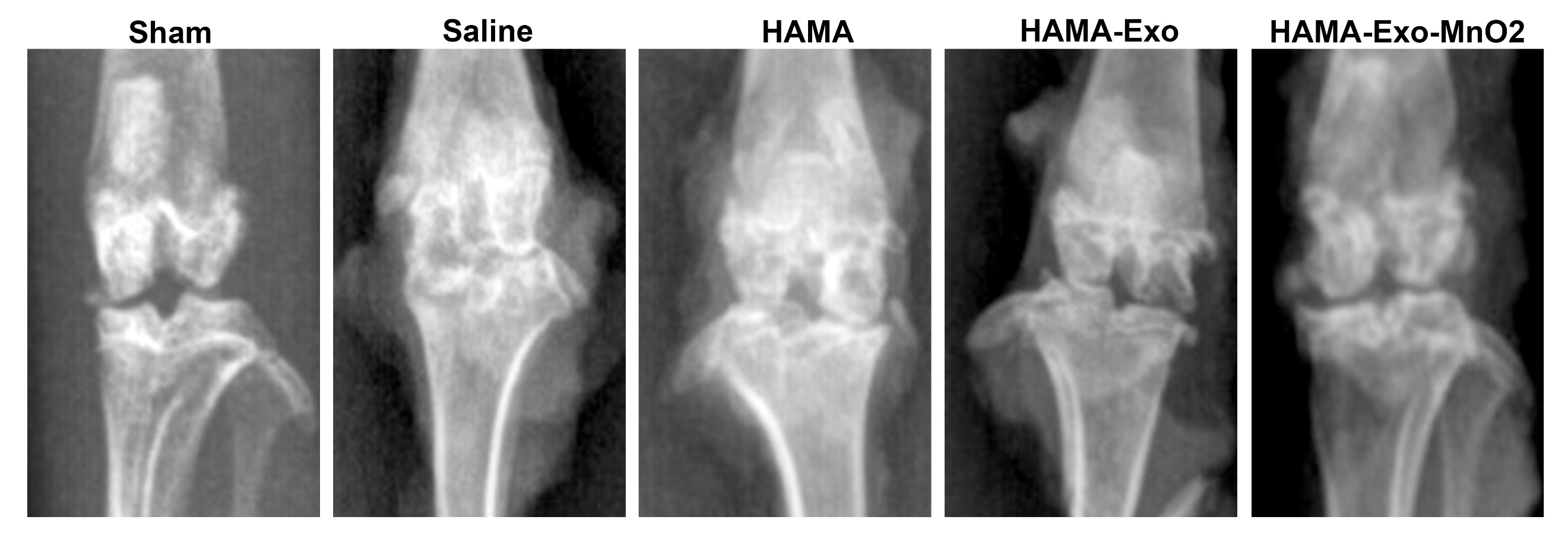


Fig. S6 Representative images of X-ray examination
